# Novel Non-Invasive Biomonitoring Using Avian Faecal Sacs Reveals Dependence of Pesticide Exposure on Field Distance

**DOI:** 10.3390/toxics14010095

**Published:** 2026-01-21

**Authors:** Moritz Meinken, Johannes Amshoff, Sascha Buchholz, Kathrin Fisch, Sebastian Fischer, Alexandra Esther

**Affiliations:** 1 Institute of Landscape Ecology, University of Münster, Heisenbergstraße 2, 48149 Münster, Germany; johannesamshoff@gmail.com (J.A.); saschabuchholz@uni-muenster.de (S.B.); s.fischer.98@web.de (S.F.); 2 Julius Kühn Institute (JKI), Institute for Ecological Chemistry, Plant Analysis and Stored Product Protection, Königin-Luise-Straße 19, 14195 Berlin, Germany; kathrin.fisch@julius-kuehn.de; 3 Julius Kühn Institute (JKI), Institute for Plant Protection in Horticulture and Urban Green, Messeweg 11/12, 38104 Braunschweig, Germany

**Keywords:** breeding birds, nest, blue tit, great tit, pesticides, caffeine

## Abstract

Pesticides remain among the most significant threats to biodiversity and natural ecosystems. Non-invasive methods, such as the analysis of bird faeces, have shown great potential for detecting pesticide exposure. In this study with a new approach, we analysed faecal sacs from nestlings of Blue tits (*Cyanistes caeruleus*) and Great tits (*Parus major*) to gain deeper insights into pesticide contamination during the breeding period. Samples were collected from three distinct sites near Münster, Germany. In total, we detected 65 substances from 57 different pesticides, as well as caffeine, with pesticides present in 16.07% of the 168 samples. Concentrations varied between species and sites and were higher for fungicides and insecticides in nests located closer to agricultural fields. While no direct effects on reproductive success were found, our results underscore the potential of faecal sac analysis as a valuable tool for spatially resolved pesticide monitoring. The novel, non-invasive approach to pesticide monitoring offers crucial exposure data on juvenile birds during their sensitive breeding period.

## 1. Introduction

Human activities have an increasingly negative impact on biodiversity [1]. Besides land-use change and microplastic, one major anthropogenic driver on terrestrial ecosystems are pesticides [2]. Their main field of application is modern agriculture, but substances are also increasingly emitted in urban environments [3,4]. Pesticides spread everywhere via air and water, so they can have unintended negative effects far from where they are used [5,6,7].

Overall, it is suspected that a large proportion of wildlife is exposed to pesticides [8]. In Central Europe, residues such as the herbicide terbuthylazine, the fungicide azoxystrobin, and the insecticide thiacloprid have been found in insects in nature reserves [9], which could lead to trophic cascade effects on birds [10,11,12]. Birds play a crucial role in ecosystems, providing a range of essential ecosystem services including predation, pollination, scavenging, seed dispersal and ecosystem engineering [13]. Furthermore, birds are highly effective bioindicators as very mobile consumers, offering valuable insights into ecosystem health and environmental change [14]. The potential risks of pesticides to birds must be evaluated separately from mammals, as the toxicity of some pesticides is higher due to the lower activity of avian metabolic enzymes [15]. Several pesticides such as insecticides [16,17], fungicides and herbicides [18,19] have been found mainly in farmland birds.

The potential of non-invasively collected faeces as an indicator for pesticide exposure have been shown [18]. However, during the different seasons, many captured individual birds lack a clear area reference, as they often change both their feeding and roosting sites. The breeding period is one of the few stages where it can be safely assumed that birds stay close to the same places—the nest area—for an extended period. Nests are particularly useful for pesticide testing, as they allow for the analysis of both adult and young birds, the latter of which tend to be easier to catch, due to their not fully developed wings. The ringing of fledglings in the nest is a commonly used method in scientific bird ringing, which establishes a straightforward sampling possibility [19].

The adult birds have a strong influence on the brood, building the nest and raising the young until they fledge [20]. In songbirds, the young are fed mostly with insects as they are high in protein and energy and therefore a particularly important source of nutrition [21]. Fledglings excrete droppings, which the adults can easily carry out of the nest thanks to a protective covering, the so-called faecal sacs. This makes the faeces less susceptible to external contamination than the droppings of adult birds [22]. In the past, faecal sacs have been successfully used for the analysis of metal exposure [23]. Since all the young of a bird pair are raised side by side in a nest, influences often affect all the young at a very early stage, which can affect their future life. The nidobiome, a concept that integrates parents, nest environments, and neonates as nest microbiome modifiers, is a fragile yet indispensable ecosystem for the reproduction and therefore the survival of bird species [20]. This highlights the importance of studying the effects of pesticides for this life period.

The most severe consequence of pesticide contamination is the death of a bird, and if it affects an adult, it has a devastating effect on the brood [24]. Furthermore, a plethora of other mostly negative effects are possible [25,26], which are complicated by natural factors influencing broods such as weather [27] and timing [28]. To assess the consequences of pesticides on breeding birds, it is necessary to determine the breeding success and other performance parameters precisely during different stages of the brood in a real-life environment.

This study selected two co-occurring species of passerine birds, the Blue tit (*Cyanistes caeruleus*) and Great tit (*Parus major*), based on their abundance and wide distribution across a range of habitats [29,30]. With the exception of the insecticide cypermethrin, which significantly impaired the weight and survival rate of nestlings, no other effects on reproduction were observed in blue tits [31,32,33]. Because research on pesticides in birds has focused primarily on agricultural areas as main source, little is known about pesticide exposure and its consequences throughout their natural habitats [8,34,35]. 

In order to improve our understanding of the impact of pesticides in natural habitats, we employed a novel, non-invasive biomonitoring technique. This involved collecting faecal sacs from blue and great tit fledglings in nest boxes situated in three different areas around Münster in Germany. We examined which pesticide substances were present and identified the factors that should be considered in future analyses. Addressing these issues is essential for achieving a comprehensive understanding of the impact of pesticides on our ecosystems.

## 2. Materials and Methods

### 2.1. Sampling

**Figure 1:**
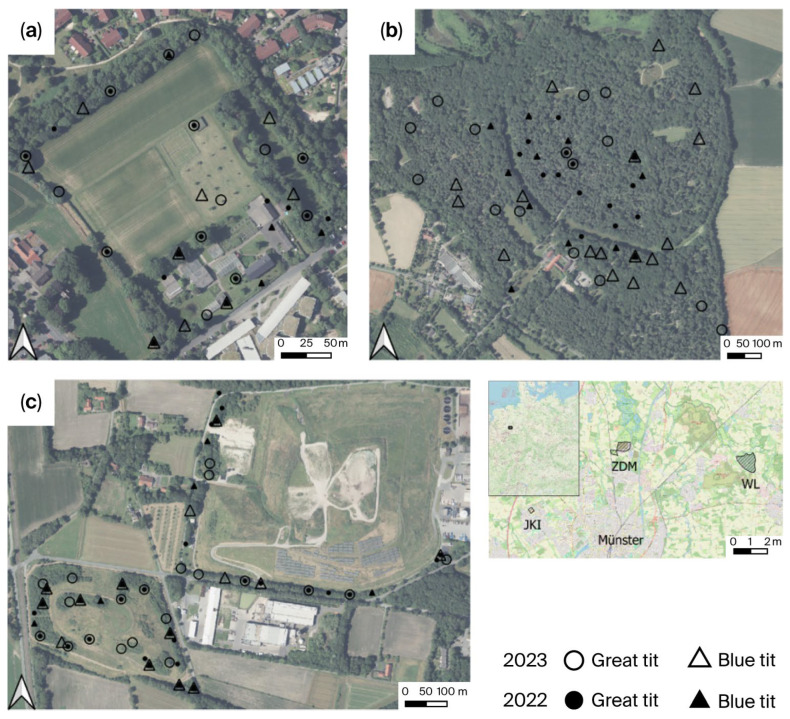
Map showing sampling points across the three study areas: (**a**) JKI, (**b**) WL and (**c**) ZDM categorised by year and species [36].

The study areas are located in and around the city of Münster in North Rhine-Westphalia, Germany. Three distinct areas with nest boxes were selected to sample Blue and Great tits nests in different land use environments (Figure 1 & Table S1): (a) JKI: A research institute with park-like grounds and cultivated fields in an urban area adjacent to agricultural land (6.58 ha), (b) WL: a mixed forest, used as a cemetery (85.60 ha), and (c) ZDM: a recultivated landfill site with mostly permanent herbaceous areas, managed by late mowing once a year (47.87 ha).

The sampling took place during the scientific bird ringing of nestlings, which is part of a programme by the Helgoland bird ringing centre. Field work was conducted during 2022 and 2023. Nest boxes were checked weekly starting from the end of April. The young birds were ringed between ages of 8 to 15 days. The breeding success for each nest box resulted from the number of young birds ringed. In addition, unfertilised eggs and dead juveniles in the nest box were counted. The two species compete for nesting facilities in areas where both species co-occur. Great tits tend to prevail in the competition for nesting sites due to their larger size. Conversely, Blue tits have been observed to have an advantage when competing for food during the breeding season [37]. Great tits typically lay 6 to 12 eggs in mid-April, with hatching commencing 13 to 15 days later. The young are fed for a further 18 to 21 days in the nest until they fledge. Blue tits lay slightly more eggs (7 to 13) with similar timing [38]. The hatching success rate varies between 82% and 98% for both species. Breeding success fluctuates from year to year due to several environmental factors but declines during the course of a breeding season [39]. Second broods are a common occurrence in both species, with third broods also occasionally observed [40]. During the breeding season, caterpillars play a significant role in providing nutrition for the young. Additionally, spiders and other insect larvae and imagines are consumed [40].

The samples were collected between 27 April and 30 May. Samples were collected during the process of ringing by placing the young on chlorine-free bleached paper bags. The sampling and ringing took under 2 min per nest depending on the number of fledglings, after which they were all immediately returned to their nest. All faecal samples from a single brood were collected in one sample tube without physical contact and were immediately cooled. Although pesticide residues persist for at least 30 days in weathered bird droppings, the samples were placed in a cool bag with ice packs for the transport to protect them from heat and direct sunlight [41]. The samples were frozen as soon as possible, first in a freezer at −20 degrees Celsius and later stored at −80 degrees Celsius until laboratory analysis.

Several factors potentially influencing the results were recorded: weight of the sample, time of sampling (day of the year and year) and location of the nest box. The distance to the next fields was measured for each nest box, while the land use within a defined radius of 50 m was also determined using the Corine Land Cover dataset (resolution of 10 m per grid cell). Every cell is assigned to one of eleven land cover classes, seven of which were present in the study area (sealed, woody needle-leaved trees, woody broadleaved deciduous trees, low-growing woody, permanent herbaceous, periodically herbaceous, non- and sparsely vegetated) [42]. QGIS was used to determine the percentage of each land cover class within the radius (50 m) of each nest box [43]. In addition, the closest distance from the nest box to the nearest field was measured using QGIS.

### 2.2. Substance Analysis

The Julius Kühn Institute for Ecological Chemistry, Plant Analysis and Stored Product Protection in Berlin prepared and analysed the samples. The faecal samples were freeze-dried and weighed upon transfer to extraction tubes. Sample preparation followed the modified QuEChERS method based on DIN EN 15662 [44] (Table S2). The internal standard was added to the samples, which were then mixed with cooled solvents (4 °C) and left to cool overnight (Table S2) [45]. The next day, the QuEChERS salt was added and vigorously shaken. The sample was then centrifuged, and a portion of the supernatant was purified using dispersive solid phase extraction (dSPE). Subsequently, an analysis was conducted utilising LC QTRAP-MS/MS [45]. Quality assurance and control samples were also analysed. A total of 108 substances were analysed. These consist of substances that are particularly toxic and environmentally harmful, as well as other substances included in the national action plan for sustainable use, such as fungicides, herbicides, insecticides and caffeine [46]. Concentrations are expressed in ng/g of the sample. Substances’ limit of detection (LOD) range between 0.0001 and 0.081 ng/g. Limits of quantification (LOQ) range between 0.001 and 4.115 ng/g.

### 2.3. Statistical Analysis

The statistical analysis was performed with R [47]. Firstly differences between the two species were analysed using the Bray-Curtis dissimilarity index of the package vegan [48]. Although the differences were significant, the observed R^2^ values were minimal. To establish a more robust foundation for subsequent analysis, the two species were therefore considered collectively.

The substances were assigned to the pesticide categories (fungicides/herbicides/insecticides) according to their field of application. As caffeine is not a pesticide, but a contaminant linked to anthropogenic influence, it was analysed separately. The metabolites and their parent compounds were included in the totals for each pesticide category.

We analysed the differences between the areas by boxplots dividing the data per area and the pesticide categories. Kruskal-Wallis tests were performed for the different pesticide categories, post-hoc tests with the Holm method provided further insights.

To further investigate the causal relationships and effects of pesticides on Blue and Great tits, we performed a series of generalised linear mixed-effects models (GLMMs) using the glmmTMB package [49]. All models were considered with the concentration of pesticides per nestbox by pesticide category and the sum of all pesticides (herbicides + fungicides + insecticides) as well as presence/absence of pesticides. Given that the samples were taken in different areas and years, they were incorporated as a random effect. The concentrations were determined by setting the zero values to LOD/2 and the values between LOD and LOQ to (LOD + LOQ)/2. This made the analysis more accurate and robust. In addition to the day of the year, the analysed independent variables included sample weight, distance to the nearest field, and the area of all land cover classes found within 50 m of the nest boxes. As the distance to the next field showed an influence around the threshold (*p* = 0.05), we used boxplots to gain a clearer picture. This was tested again using a Kruskal–Wallis test and a post-hoc test with the Holm method.

Moreover, an analysis was conducted to ascertain the effect of pesticide contamination (concentrations and presence/absence) on the total number of young birds, the number of unfertilised eggs, and the number of dead juveniles. In all models the best model fit was chosen using the Akaike Information Criterion (AIC ≤ 2). All graphical statistic elements were generated using ggplot2 with the help of ggeffects [50,51]. The full details of all GLMMs are provided in the supplement (Table S6).

## 3. Results

### 3.1. Residues in Blue and Great Tits

In total, 168 samples (Blue tit = 73, Great tit = 95) from the three areas (JKI = 43, WL = 57, ZDM = 68) contained 65 substances (39 herbicides, 17 fungicides, 8 insecticides and caffeine) of 57 pesticides and 8 corresponding metabolites. Significant differences in residues were found between the two species (F = 6.09, R^2^ = 0.035, *p* = 0.007, Bray-Curtis test). There were differences in occurrence of pesticide substances between the three areas (Table S3). In JKI, 45 substances were identified in 14 samples (44 > LOQ). In WL, 16 substances were detected in nine samples, while in ZDM, 29 substances were identified in four samples. Overall, pesticide substances were identified in 16.07% of the samples. Most of the loads could be quantified, except for two values of the fungicide fenpropimorph, and one for the herbicides chlorotoluron and diflufenican respectively, as the concentrations were below the limit of quantification (LOQ) (Table S3). The concentrations reached a maximum in dimefuron with 208.92 ng/g for herbicides, in thiacloprid-amid with 206.46 ng/g for insecticides and in dimethomorph with 194.92 ng/g for fungicides (Table 1, Table S4).

Caffeine, which was identified in 4.76% of the samples, reached a maximum of 1287.70 ng/g. This would represent 1.29 mg/kg body weight, however most of the pesticide values found do not exceed 0.2 mg/kg body weight of a bird (Table S4).

### 3.2. Influencing Factors

**Figure 2:**
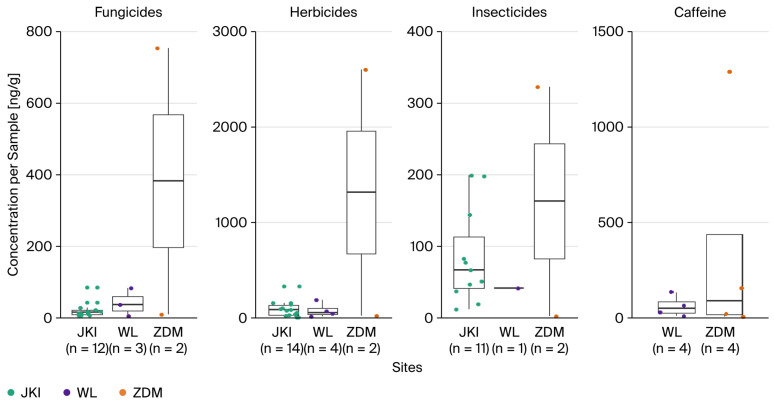
Boxplots showing the concentrations of different substances (fungicides, herbicides, insecticides, and caffeine) in contaminated samples across the three study areas.

The concentrations of fungicides in contaminated samples (median: 13.88 (JKI) vs. 34.17 (WL) vs. 380.81 (ZDM) ng/g), herbicides (79.21 vs. 54.26 vs. 1310.84 ng/g) and insecticides (66.69 vs. 40.9 vs. 162.14 ng/g) exhibited no significant differences (Kruskal-Wallis test) between the sites (Figure 2). Insecticides were almost all detected in JKI, where caffeine (0 vs. 45.95 vs. 85.93 ng/g) was not detected at all. There were no differences in sample weight, but there were differences in sampling day between the areas (H = 8.53, df = 2, *p* < 0.05, Kruskal-Wallis test), which were significantly earlier in JKI (mean: 13 May) than in ZDM (17 May, Z = −2.88, *p* < 0.05, post-hoc test), though not WL (14 May).

**Figure 3:**
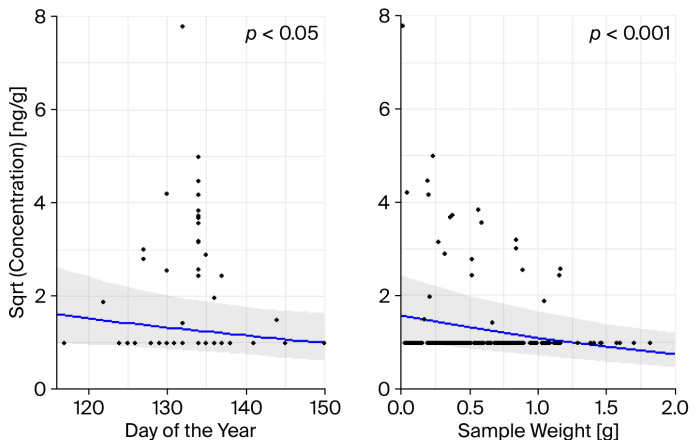
Effect plots of the generalised linear mixed model (GLMMs) showing the relationships between the day of the year and the sample weight with the total concentration of fungicides, herbicides, and insecticides for each sample. The regression line is presented with 95% confidence intervals, which account for the random effects of year and area (Table S6).

The total concentration of fungicides, herbicides, and insecticides was found to be significantly negatively affected by the day of the year (*p* < 0.05) and sample weight (*p* < 0.001) (Figure 3).

**Figure 4:**
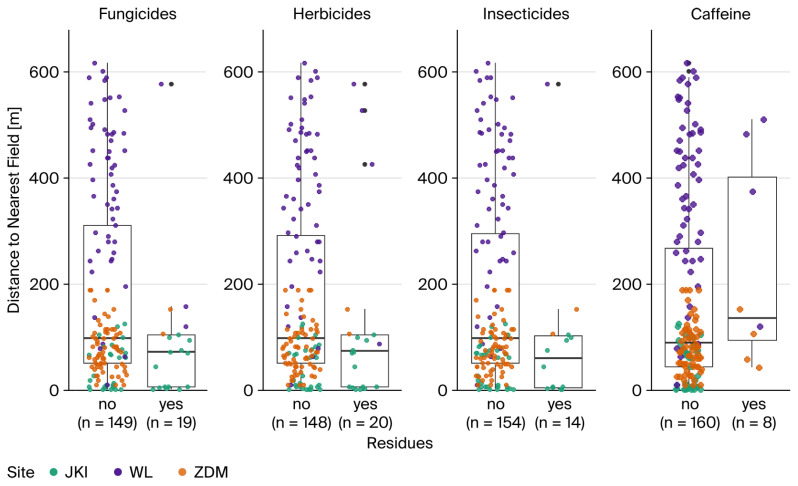
Boxplots showing the distance to the nearest field for all samples, divided into residues detection for fungicides, herbicides, insecticides, and caffeine.

The fledglings contaminated with fungicides were raised in nest boxes significantly closer to the next field than the uncontaminated ones (median: 71.3 vs. 97.7, W = 1864, *p* < 0.05, Wilcoxon test). The same could be observed for insecticides (60.4 vs. 97.6, W = 1465, *p* < 0.05). However, caffeine (136.0 vs. 90.0, W = 477, *p* = 0.23) and herbicide contamination (73.5 vs. 98.4, W = 1847, *p* = 0.07) showed no distinct relation with the distance to the nearest field (Figure 4).

The exposure of fledglings to pesticides did not result in a significant change in the number of fledglings, the number of unfertilised eggs or the number of dead juveniles in the nests (Table S6).

## 4. Discussion

In total, 65 substances (57 pesticides and eight metabolites) were detected in 16.07% of the samples and in up to 208.92 ng/g per pesticide with significant differences between the two species and differences between the areas. Caffeine exhibited an appearance pattern that was distinct from the other substances with residues found in eight broods up to 1287.7 ng/g. There was no significant influence of landscape composition on pesticide contamination. The most influential factors on pesticide contamination were sample size and the distance to the nearest field. We found no effects of the pesticide residues on the broods’ performance.

### 4.1. Residues in Blue and Great Tits

Several substances were identified which present a significant toxicological risk to birds. These include the banned herbicide prosulfocarb [8], persistent neonicotinoids such as thiamethoxam, thiacloprid [10] and imidacloprid, which showed adverse effects in Northern Bobwhites (*Colinus virgianus*) such as eggshell thinning and bodyweight reduction [52]. Neonicotinoids are also known to reduce feeding and accumulation of body mass and fat stores [53]. Other persistent substances, including the herbicides dimefuron and flupyrsulfuron-methyl, were found in elevated concentrations that have been prohibited for some time.

The concentrations identified in this study do not exceed the established limits for the substances, including the acute oral LD50 [54]. However, due to missing specified toxicity thresholds for songbirds, the final interpretation of concentrations and their ecological risk is not possible. Due to the pooled samples, it is possible to underestimate individual contaminations and risks to highly exposed juveniles. The concentrations obtained are merely a proxy for the intake as substances can be absorbed and distributed to tissues [55]. Furthermore, it should be noted that the faecal samples collected may not provide a comprehensive representation of the entire development period from birth to maturity, as they are only collected at specific points in time. Among other factors, the nutrition of the fledglings is dependent on the habitat structure, brood timing and nestling age, resulting in a variable individual nutrition profile for each brood [56,57,58]. Consequently, it can be concluded that the concentrations of pesticides obtained are merely a snapshot of the total load to which the young tits are exposed.

Caffeine enters the environment through human activities, primarily via wastewater and sewage [59]. Contamination increases in areas with a higher human density, which consequently can lead to a higher consumption rate by birds [60]. Its adverse effects include oxidative stress, metabolic disruption, and reproductive impacts [59]. Although the focus of caffeine research is primarily on wastewater and marine coastal systems, the impact on terrestrial animals should not be overlooked [59]. Approximately 35% of reported environmental concentrations exceed predicted no-effect levels [60]. Small concentrations (<50 mg/kg) of caffeine seem to increase growth in chicken (*Gallus domesticus*) [61], but also cause pulmonary hypertension syndrome [62]. At 366 mg/kg [61], the LD50 for feral pigeons (*Columba livia domestica*) is relatively low compared to pesticides that are still in use [63].

### 4.2. Influencing Factors

The areas analysed do not differ sufficiently to draw definitive conclusions about the origin and entry routes of the different pesticides. It is noteworthy that the JKI, which has the most substantial evidence of herbicides, fungicides and insecticides, is the only area with a proportion of periodically herbaceous areas in the vicinity of the nest boxes. However, in view of the wider suburban environment of the area, other routes of entry for pesticides are also possible [4]. The resolution of the employed landscape classification is low (10 m grid) when analysing a 50 m radius around the nest boxes. Higher resolution products may offer enhanced capacity to account for actual fine-scale landscape composition, thereby more effectively highlighting differences. The landscape classes utilised in this study were predetermined by the Corine Land Cover classification system. The creation of classes based on the landscape present in the study area has the potential to enhance the precision through more sensible classification. The employment of drones in conjunction with machine learning landscape classification is a promising methodology for conducting fine-scale landscape analysis [64,65]. As parent birds can forage more than 50 m away from the nest, the set radius may not capture the full foraging range [66]. This is dependent on food supply and therefore habitat structure. However, the likelihood of a bird foraging decreases with distance from the nest box during the breeding season, meaning the nearby habitat plays the most important role [66].

Lower concentrations were found in heavier samples. The deposition of pesticides may be analogous to metals, which exhibit concentrations that vary between the urate and faeces parts of faecal sacs [23]. Naturally, every faecal sac is different in size, but also in urate and faeces proportion with their differing loads. This would mean that the proportions of urate and faeces, as well as their individual contamination profiles, would have to be quantified in each faecal sample by laboratory analysis. Only then would it be possible to compare different samples.

Concentrations were found to be higher the earlier the samples were taken (Figure 3). The young birds must have ingested the pesticides. Most likely, this occurred through the insects they were fed. Temporal variability can possibly be explained by differences in insect exposure or in the locations where the insects were caught [9]. Furthermore, Great tits tend to collect more insects from the ground during the early stages of the breeding season; in the later stages they forage mostly among higher vegetation [67].

The agricultural land surrounding Münster is primarily used for the cultivation of cereal crops, maize, and potatoes [68]. Most of the substances found can be traced back to agricultural activities, especially to those frequently cultivated crops (Table S5). The significant correlation between the substances with the distance to the next field suggests that a substantial proportion of the pesticides identified originate from agricultural activities. Numerous other studies have already linked contamination of birds with agricultural pesticides [8,17,18]. However, those species analysed are frequently representative of the typical avifauna of agricultural landscapes [35]. In contrast, Blue and Great tits only sporadically visit these structures for foraging purposes during the breeding season [69]. Naturally, they tend to follow food sources, demonstrating a preference for organic over conventional agricultural practices [69]. Nevertheless, it can be reasonably assumed that breeding that takes place near arable land (as in JKI) will result in the birds foraging in or near the field with greater frequency. Consequently, these birds will be more susceptible to contamination by pesticides. Broods that are situated at a greater distance from the field may, for instance, become contaminated by pesticide-laden insects that carry the substances from the field into the surrounding area [9].

However, non-agricultural sources, such as horticulture and roadside application (Table S5), are also possible in addition to atmospheric deposition [70]. Materials utilised in the construction of the nest have the potential to introduce pesticides into the nidobiome. Insecticides were found in all Blue and Great tit nests in a study from the UK [71]. For instance, imidacloprid, thiacloprid and thiamethoxam are brought into the nest with fur from companion animals used for the nest construction [71]. Consequently, juvenile birds in the nest are directly exposed to these substances.

No significant effects on reproductive success, as measured by three parameters, were observed. In previous studies substances, that we couldn’t detect in this study, like the insecticide cypermethrin had significant negative influence on nestling weight and survival, while the insecticide malathion and the fungicide propiconazole had no effect of breeding performance parameters [31,32,33]. Additionally, we were unable to consider the possibility of sublethal effects, such as behavioural changes, immunosuppression or illness, in the study species due to pesticide exposure.

No significant effects on reproductive success (i.e., unfertilised eggs, number of young birds ringed and dead juveniles) were found. Nevertheless, sublethal effects (e.g., behavioural changes, immunosuppression) or long-term consequences at the population level are possible. However, the necessary information on this is lacking.

## 5. Conclusions

The novel, non-invasive approach to pesticide monitoring reveals the critical exposure of juvenile birds during the sensitive breeding period. It provides an opportunity to analyse the influence of the surrounding environment during an important time. Our findings indicate the exposure of juvenile birds by detecting 65 substances (57 pesticides and eight metabolites) and linking exposure to distance from agricultural fields, based on faecal sac analysis. It shows that pesticides are present in the food web. Furthermore, we detected pesticides in all areas, despite a diverse range of land uses, which highlights the widespread exposure to these substances. The selection of monotypic study areas with distinct habitat types is crucial for identifying the entry routes of contaminants. Close monitoring of the brood, together with consideration of a broad set of ecological variables, allows potential impacts on nestling development and survival to be assessed. Given that caffeine has been found in faecal samples, future research should investigate its possible entry routes into the environment and its ecological implications.

## Figures and Tables

**Table 1:** Concentrations of detected pesticides and their metabolites, in more than 2% of the faecal sac samples from Great and Blue tits (see Table S4 for findings less than 2%), represented by detection frequency per species (%), as well as median and maximum (all in ng/g) of the concentrations found.

	Blue Tit (n = 73)			Great Tit (n = 95)		
Substance	det. Freq. [%]	Median [ng/g]	Max [ng/g]	det. Freq [%]	Median [ng/g]	Max [ng/g]
Caffeine	6.85	65.71	152.45	3.16	19.41	1287.70
Carbendazim, fungicide	5.48	5.48	9.63	8.42	6.07	15.02
Chlorantraniliprole, insecticide	4.11	19.60	21.69	7.37	11.11	44.72
Chloridazon, herbicide	1.37	1.89	1.89	6.32	12.03	31.93
Diflufenican, herbicide	4.11	8.76	14.18	5.26	2.46	89.85
Dimefuron, herbicide	5.48	12.46	25.61	10.53	5.80	208.92
Diuron, herbicide	4.11	8.04	17.14	4.21	4.49	207.23
Fenuron, herbicide	2.74	13.43	20.92	5.26	4.20	11.33
Fluopicolide, fungicide	5.48	11.17	36.38	6.32	2.65	135.70
Flupyrsulfuron-methyl, herbicide	4.11	35.34	74.18	7.37	30.86	92.36
Foramsulfuron, herbicide	4.11	1.96	31.67	1.05	5.49	5.49
Imidacloprid, insecticide	1.37	3.04	3.04	3.16	1.42	18.11
Methiocarb, insecticide	4.11	55.19	111.53	7.37	27.20	111.19
Prosulfuron, herbicide	1.37	1.94	1.94	6.32	4.53	171.08
Terbuthylazine-2-hydroxy-desethyl, herbicide	2.74	5.66	6.22	3.16	12.45	140.46
Terbuthylazine-desethyl, herbicide	4.11	7.60	31.04	5.26	9.11	134.92
Thiacloprid, insecticide	2.74	18.27	26.53	6.32	10.55	36.22
*Thiacloprid-Amid, insecticide*	*4.11*	*16.43*	*40.90*	*8.42*	*5.02*	*206.46*
Zoxamide, fungicide	4.11	6.84	7.88	6.32	6.90	14.08

## Data Availability

The original contributions presented in this study are included in the article/Supplementary Material. Further inquiries can be directed to the corresponding authors.

## Supplementary Materials

The following supporting information can be downloaded at: https://www.mdpi.com/article/10.3390/toxics14010095/s1, Table S1: mean land use; Table S2: analytic procedure; Table S3: substances detected; Table S4: substances per species; Table S5: application, load etc.; Table S6: models.
